# Sin nombre virus in deer mice captured inside homes, southwestern Montana.

**DOI:** 10.3201/eid0604.000411

**Published:** 2000

**Authors:** A J Kuenzi, R J Douglass, C W Bond

**Affiliations:** Department of Biology, Montana Tech of the University of Montana, Butte, Montana 59701, USA. akuenzi@mtech.edu

## Abstract

From 1996 through 1999, 35 deer mice (Peromyscus maniculatus) were captured in 25 urban and suburban homes in southwestern Montana. Mice were captured throughout the year except for January; seven mice (20%) from seven (28%) of the homes were seropositive for Sin Nombre virus. The infected mice were mostly adult males captured in the spring and fall.


### Sin Nombre Virus in Deer Mice Captured Inside Homes, Southwestern Montana

**Amy J. Kuenzi,* Richard J. Douglass,* and Clifford W. Bond†**
*Montana Tech, Butte, Montana, USA; and
†Montana State University, Bozeman, Montana, USA

From 1996 through 1999, 35 deer mice (*Peromyscus maniculatus*) were captured in 25 urban and suburban homes in southwestern Montana. Mice were captured throughout the year except for January; seven mice (20%) from seven (28%) of the homes were seropositive for Sin Nombre virus. The infected mice were mostly adult males captured in the spring and fall.

Hantavirus pulmonary syndrome (HPS), first recognized in 1993, is caused by several related viruses in the genus *Hantavirus*. The primary reservoir of one of these, Sin Nombre virus (SNV), is the deer mouse, *Peromyscus maniculatus*. Deer mice adapt to a variety of habitats and are found across most of the United States; their presence in and around homes has been implicated as a risk factor for HPS. Case-control studies of the original outbreak in the Four Corners region of the southwestern United States found that hantavirus infection prevalence (as indicated by the presence of antibody) in deer mice in and around urban and rural homes was 27.5% to 32.5%, with no significant difference in prevalence between homes of case and control patients. The prevalence of hantavirus infection in deer mice that invade homes in other parts of the United States has not been determined because of the difficulty in obtaining samples from occupied homes. Because the rates of deer mouse infestation in urban and suburban homes are low, extensive random sampling is both nonproductive and disruptive to home owners.

We describe the temporal patterns and age and sex characteristics of deer mice in urban (residential subdivisions) and suburban (>2-ha lots near the edge of town) homes in a nonoutbreak area and determine the prevalence of infection in deer mice captured inside these homes. This study was conducted in and near Butte (population 30,000), Silver Bow County, Montana, USA. Although 12 cases of HPS, with three deaths, have been documented in Montana, none occurred in this area of the state.

Address for correspondence: Amy J. Kuenzi, Department of Biology, Montana Tech of the University of Montana, Butte, MT 59701, USA; fax: 406-496-4135; e-mail: akuenzi@mtech.edu.

### The Study

From November 1996 through September 1999, we provided Sherman live traps to persons who contacted us or local exterminators with complaints of rodent infestation in their homes. Traps containing animals were placed in plastic bags and brought to us for processing according to the protocols of Mills et al.(1). Sex and age were recorded for each captured mouse. Age was based on the following weight categories: <14 g juvenile, 14 to 17 g subadult, and >17 g adult (2). Mice were examined for injuries (scarred tails or torn or nicked ears) possibly indicating fights. Blood samples were collected from the retroorbital sinus of each animal with a heparinized capillary tube and were stored on dry ice until transferred to –70°C freezers for storage. Serologic testing was conducted at Montana State University, Bozeman, Montana. Samples of whole blood were tested for antibody reactive with SNV recombinant nucleocapsid protein by enzyme-linked immunosorbent assay (3).

We captured 35 *P. maniculatus* from 25 homes (Table). Seven mice from seven of the homes were seropositive for antibodies to SNV, an overall prevalence of 20%. Sex and age ratios were similar between seropositive and negative deer mice. More adult than subadult and juvenile and more male than female mice were captured inside homes. Most seropositive mice (71.4%) were male, and >50% of these were adult. One seropositive mouse (14.3%) had evidence of injuries. *P. maniculatus* were captured inside homes throughout the year with the exception of January (Figure). However, seropositive mice were captured only in the spring and fall. Juvenile deer mice were captured only in September and October.

Table. Distribution of antibody-positive and -negative deer mice, by sex, age,[1] and evidence of injuries

| Characteristic | Antibody positive no. (%) | Antibody negative no. (%) | Total (%) |
|---|---|---|---|
| Sex | | | |
| Male | 5 (71.4) | 18 (64.3) | 23 (65.7) |
| Female | 2 (28.6) | 10 (35.7) | 12 (34.3) |
| Total | 7 | 28 | 35 |
| Age | | | |
| Juvenile | 1 (14.3) | 1 (3.7) | 2 (5.9) |
| Subadult | 2 (28.6) | 11 (40.7) | 13 (38.2) |
| Adult | 4 (57.1) | 15 (55.6) | 19 (55.9) |
| Total | 7 | 27 | 34 |
| Injuries | | | |
| Yes | 1 (14.3) | 3 (10.7) | 4 (11.4) |
| No | 6 (85.7) | 25 (89.3) | 31 (88.6) |
| Total | 7 | 28 | 35 |

[1]Age information was not collected for one mouse.

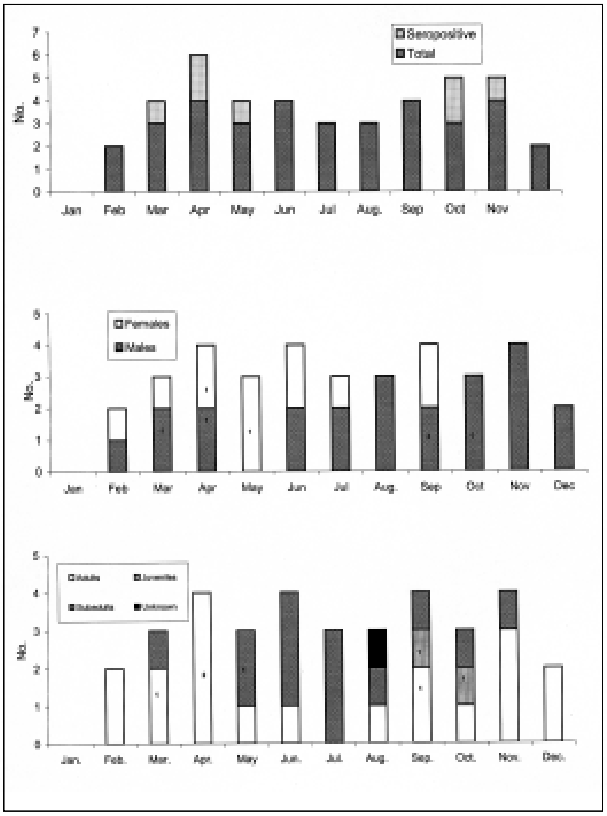

Figure. Temporal patterns of deer mice trapped in urban and suburban homes, November 1996 to September 1999. Numbers inside bars indicate seropositive mice.

### Conclusions

The overall prevalence of antibodies reactive with SNV antigen in deer mice invading homes was 20%. This prevalence is lower than that of deer mice captured in and around homes during the 1993 Four Corners outbreak but higher than the overall prevalences of sylvatic deer mice populations in Montana, Colorado, Kansas, national parks in eastern and central United States, and major biotic communities in the southwestern United States.

Most of the mice invading homes were adult males. Prevalence of infection (as indicated by antibody) was higher in male and sexually mature rodents, as in other studies (4-7). Although we detected few direct signs of aggressive encounters in individual mice, others have reported a higher incidence of injuries in infected mice (4,8), which implicates fighting as a mode of transmission.

Deer mice were trapped inside homes throughout the year except for January, which indicates that mice do not necessarily invade homes more readily during cold weather. Seropositive mice, however, were captured only during spring and fall, which indicates a higher prevalence of infection in deer mice populations during those times. Sylvatic populations of *P. boylii* in northern Arizona show a bimodal pattern of hantavirus transmission, with peaks in the spring and fall (6). Cases of human infection in the Four Corners region have a spring-summer seasonal pattern (9,10). However, peridomestic populations of deer mice from two study sites near Butte, Montana, had no distinct seasonal patterns of transmission of hantavirus infection (unpub. data, Douglass and Kuenzi). The seasonal patterns we found in deer mice captured in homes may be an artifact of sample size.

Infestation of homes by deer mice is not restricted to rural environments. SNV infection may be as prevalent in deer mice captured in homes in urban and suburban environments as in populations in sylvatic habitats. Urban and suburban homeowners are not exempt from the risk for hantavirus infection and should follow recommendations for risk reduction (11).

### Acknowledgments

We thank C. Younce, T. Wilson, S. Douglass, D. White, Jr., and many homeowners for their assistance; B. Peterson for laboratory support; and J.N. Mills for review of the manuscript.

Funding for this study was provided by the Centers for Disease Control and Prevention, Atlanta, Georgia.

Dr. Kuenzi is an assistant research professor at Montana Tech of the University of Montana. Her research focuses on small mammal population ecology.

### References

1. Mills JN, Yates TL, Childs JE, Parmenter RR, Ksiazek TG, Rollin PE, et al. Guidelines for working with rodents potentially infected with hantavirus. J Mammal 1995;76:716-22.
2. Fairbairn DJ. The spring decline in deer mice: death or dispersal? Can J Zool 1977;55:84-92.
3. Feldmann H, Sanchez A, Morzunov S, Spiropoulou CF, Rollin PE, Ksiazek TG, et al. Utilization of autopsy RNA for the synthesis of the nucleocapsid antigen of a newly recognized virus associated with hantavirus pulmonary syndrome. Virus Res 1993;30:351-67.
4. Calisher CH, Sweeney W, Mills JN, Beaty BJ. Natural history of Sin Nombre virus in western Colorado. Emerg Infect Dis 1999;5:126-34.
5. Mills JN, Ksiazek TG, Ellis BA, Rollin PE, Nichol ST, Yates TL, et al. Patterns of association with host and habitat: antibody reactive with Sin Nombre virus in small mammals in the major biotic communities of the southwestern United States. Am J Trop Med Hyg 1997;56:273-84.
6. Abbott KD, Ksiazek TG, Mills JN. Long-term hantavirus persistence in rodent populations in central Arizona. Emerg Infect Dis 1999;5:102-12.
7. Kuenzi AJ, Morrison ML, Swann DE, Hardy PC, Downard GT. A longitudinal study of Sin Nombre virus prevalence in rodents, southeastern Arizona. Emerg Infect Dis 1999;5:113-7.
8. Childs JE, Glass GE, Korch GW, LeDuc JW. Prospective seroepidemiology of hantaviruses and population dynamics of small mammal communities of Baltimore, Maryland. Am J Trop Med Hyg 1987;37:648-62.
9. Khan AS, Khabbaz RF, Armstrong LR, Holman RC, Bauer SP, Graber J, et al. Hantavirus pulmonary syndrome: the first 100 U.S. cases. J Infect Dis 1996;173:1297-303.
10. Engelthaler DM, Mosley DG, Cheek JE, Levy CE, Komatsu KK, Ettestad P, et al. Climatic and environmental patterns associated with hantavirus pulmonary syndrome, Four Corners region, United States. Emerg Infect Dis 1999;5:87-94.
11. Centers for Disease Control and Prevention. Hantavirus infection—southwestern United States: interim recommendations for risk reduction. MMWR Morb Mortal Wkly Rep 1993;42(RR-11):1-13.
